# Registered nurses’ experience as disaster preparedness coordinators during a major incident: A qualitative study

**DOI:** 10.1002/nop2.1066

**Published:** 2021-09-21

**Authors:** Jason P. Murphy, Anna Hörberg, Monica Rådestad, Lisa Kurland, Anders Rüter, Maria Jirwe

**Affiliations:** ^1^ Department of Clinical Science and Education Karolinska Institutet Stockholm Sweden; ^2^ Department of Health Promoting Science Sophiahemmet University Stockholm Sweden; ^3^ Department of Health and Welfare Dalarna University Falun Sweden; ^4^ Department of Medical Sciences Örebro University Örebro Sweden; ^5^ Department of Health Sciences Red Cross University College Stockholm Sweden; ^6^ Department of Neurobiology, Care Sciences and Society Karolinska Institutet Huddinge Sweden

**Keywords:** clinical decision‐making, disaster nursing, disaster planning, major incident, management

## Abstract

**Aims:**

To explore registered nurses’ experiences as disaster preparedness coordinators of hospital incident command groups’ during a major incident.

**Design:**

A qualitative descriptive design using semi‐structured interview.

**Methods:**

This was a qualitative study based on one focus group discussion and six individual follow‐up interviews. Participants were registered nurses in their capacity as disaster preparedness coordinators with experience from Major Incident simulations and a real‐life Major Incident. The interviews were transcribed verbatim and analysed using content analysis. The COREQ checklist was used for reporting the findings.

**Results:**

The analysis of data generated the main category: *Expectations, previous experience and uncertainty affect hospital incident command group response during a Major Incident* and three categories, (I) *Gaining situational awareness* (containing two subcategories), (II) *Transitioning to management* (containing three subcategories) and (III) *Actions taken during uncertainty* (containing two subcategories).

## 1 INTRODUCTION

Major Incidents (MIs) often require urgent medical response to mitigate morbidity and mortality (Kaj et al., 2006). An essential part of this medical response is adequate leadership preparedness, decision‐making ability and timely response (Hooker et al., 2019; The Joint Commission, 2017). Registered nurses (RNs) in disaster management roles such as disaster preparedness coordinators (DPCs) have a profound impact on patient safety and outcome during this urgent first medical response.

### 1.1 Background

Hospital response during an MI is dependent upon several factors, such as organization, disaster plans, training, and incident command structure (Bahrami et al., 2020; Djalali et al., 2013; Ripoll Gallardo et al., 2020). Well‐prepared hospitals may mitigate the effects of an MI (Chuang et al., 2019; Naser et al., 2018). During MIs, hospital incident command groups (HICGs) are activated to provide leadership and strategic decision‐making (Djalali et al., 2015; Schultz & Koenig, 2006). The HICGs, consisting of an incident commander and representatives from key departments within the hospital, are responsible for providing strategic leadership (Djalali et al., 2015). Strategic leadership requires the ability to make appropriate decisions based on scarce, and possibly unreliable information (Chuang et al., 2019; Wallenius et al., 2019).

Registered nurses holding the position as DPCs in the study setting are essential strategic managerial parts of hospitals’ overall disaster preparedness and response as members of the HICGs (National Board of Health & Welfare, 2013). DPCs form and revise disaster preparedness plans, initiate and plan exercises and participate in the evaluation of hospital preparedness (The National Board of Health & Welfare, 2013). While evaluation of disaster preparedness is vital, there is a lack of consensus on a methodology for evaluating HICG preparedness and response.

Previous studies have assessed components of HICG preparedness, that is, command structure and functional capacity (Djalali et al., 2013; Ingrassia et al., 2016; Kaji & Lewis, 2006; Woyessa et al., 2020). In addition, there is evidence that HICG preparedness can be evaluated during simulation exercises using measurable indicators (Murphy et al., 2020; Ruter et al., 2016). Previous research suggests that a lack of situational awareness may be a barrier to effective disaster response (Murphy et al., 2020). However, little is known about factors affecting HICGs’ response during an MI (Wallenius et al., 2019). Therefore, this study aims to explore registered nurses’ experiences as disaster preparedness coordinators (DPC) of hospital incident command groups during an MI.

## 2 METHOD

### 2.1 Design

A qualitative descriptive design (guided by the COREQ checklist) with semi‐structured interviews was used.

### 2.2 Setting and participants

On 7 April 2017, a major incident occurred in Stockholm, Sweden (Swedish Civil Contingencies Agency, 2018). Regional and local disaster plans were activated as were the disaster plans for all six major hospitals which entailed the establishment of HICG. All participating hospitals have the same disaster preparedness structures and followed regional disaster medicine preparedness guidelines as stipulated (National Board of Health & Welfare, 2013).

A purposive sampling technique was used. The inclusion criteria were DPCs who were operative in the HICG during both the MI in 2017 and the MI simulations in 2016 (Murphy et al., 2020). In addition, DPCs were to have aided in emergency and disaster planning and training per their role description. The participants (*n* = 6) representing 100% of the study population were all DPCs at the emergency hospitals who were affected by the MI in 2017 and therefore activated their disaster plan (five DPCs and one assistant DPC) (Table 1). The reason for including the assistant DPC from one of the hospitals was that the DPC at this specific hospital was part of the research team.

**TABLE 1:** Description of the participants *n* = 6

Gender (female/male)	6 (100%) /0 (0%)	
	Mean (years)	Range (years)
Age	50	41–62
Years as disaster preparedness coordinator	5	2–11
Years as registered nurses	27	13–40

Information power was assessed by the criteria as stipulated: study aim, sample specificity, quality of dialog and analysis strategy (Malterud et al., 2016). The study aim in the present study was narrow and required a smaller sample (Malterud et al., 2016). The sample specificity was met through recruitment of participants having specific expertise, knowledge and experience, as they pertain to the aim (requiring a smaller sample). Before undertaking the study, considering the amount of knowledge the participants had concerning the specific aim, it was determined that information power would be achieved and sample size would be relevant, although only six participants could provide the knowledge we wanted to explore (Malterud et al., 2016).

### 2.3 Data collection

Semi‐structured interviews were conducted (Patton, 2002) and interview guide developed by JM, MR, and MJ. MR was employed as a DPC during the simulations and terror attack providing expert opinion concerning the type of questions to be included. JM had previously established contact with the DPCs evaluating HICG (Murphy et al., 2020). Data collection was conducted in two phases. The first phase consisted of a focus group discussion (FGD) conducted by JM with MJ facilitating and taking notes, on 11 June 2018. The FGD began with the aid of two pictures, one depicting the simulations exercises in 2016 and the other depicted the scene of the terror attack in 2017 with the semi‐truck embedded in the department store, accompanied by the question, “It's 14.53 on Friday the 7 April 2017. News of a suspected terror attack has just been broadcasted. What were your initial thoughts?”. The interview guide contained open‐ended questions formulated according to the aim, such as, “What is your opinion of the hospital incident command group's ability to adequately manage a Major Incident”. Follow‐up questions, such as “can you tell me more about that?” and “what do you think needs to be developed and why” were utilized. Time constraints limited the FGD to 52 min and was ended before all questions were thoroughly explored and therefore a second phase was added, which consisted of individual follow‐up telephone interviews with the same participants using the same interview guide with all participants. Those interviews were conducted by JM, with 60 min allocated for the interviews. The individual interviews ranged from 3–8 min. In addition, the quality of dialog was assessed and deemed to be strong due to the interconnected criteria: knowledge and skills of the interviewer, the participants’ ability to effectively articulate and the chemistry between the participants and the interviewer. This facilitated clear and effective communication.

### 2.4 Data analysis

Inductive content analysis was used. To ensure trustworthiness, the analysis scheme described by Elo et al. was used following the three phases: (I) preparation, (II) organization and reporting (III) (Elo et al., 2014). The interviews were digitally recorded and transcribed verbatim by JM. Thereafter, as part of the preparation phase, the transcribed text was read while listening to the audio recordings multiple times to ensure an overarching understanding of the material. To minimize subjectivity and potential bias, and to increase the trustworthiness of data, investigator triangulation was used (Denzin, 1978). In the organization phase, open coding was done and discussed between JM, AH, MR and MJ. The codes were grouped, and categories were created which were then grouped under higher order headings (Table 2). In the reporting phase, the process went back and forth between codes, subcategories and categories until consensus was reached between JM, AH, MR and MJ. The analysis was confirmed by LK and AR. The design and analysis strategy were evaluated by an external professor with extensive experience in qualitative methods. Throughout the analysis process, the research group systematically reflected on the criteria for information power. The conclusion was that due to the narrow aim, sample specificity, analysis strategy and quality of dialog, adequate information power was obtained. Quotations are referred to based on the interview source, that is, focus group discussion (fgd) or follow‐up interview (fi) to ensure the participants’ confidentiality.

**TABLE 2:** Example from the analysis process

Meaning unit	Condensed unit	Code	Subcategory	Category
We had an incident commander who was not trained for this but was allowed to continue because he has gotten the HICG up and running with assistance from all of us others, but there were no formal staff briefings	Incident commander who was not trained. No staff briefings	Lack of staff briefings	Struggling with staff briefings	Transitioning to management

### 2.5 Ethical considerations

All the participants were informed of the study in written and oral form and written permission was granted. Participants were informed that participation was voluntary and could be withdrawn without consequences. Furthermore, confidentiality was guaranteed. Research Ethics Committee approval was received by the Regional Ethical Review Board number 2016/1530‐31/5.

## 3 RESULTS

**FIGURE 1:**
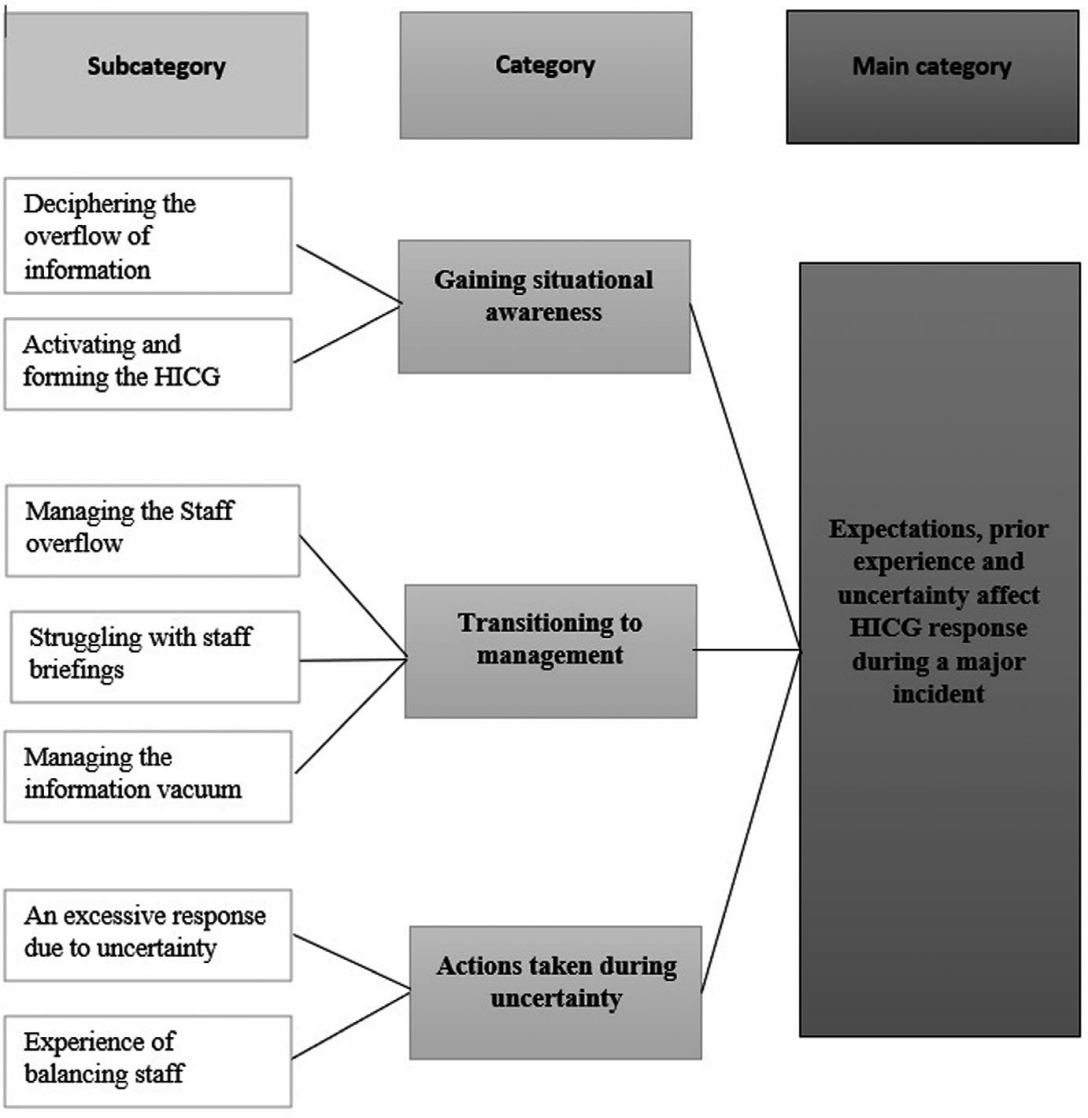
Results showing subcategories, categories, and main category

The exploration of the RN’ experiences as disaster preparedness coordinators’ (DPC) during a Major Incident, resulted in the main category, *Expectations, previous experience and uncertainty affect HICG’ response during a Major Incident* and three categories (I) *Gaining situational awareness* (containing two subcategories), (II) *Transitioning to management* (containing three subcategories) and (III) *Actions taken during uncertainty* (containing two subcategories) (Figure 1).

### 3.1 Expectations, previous experience, and uncertainty affect HICG’ response during a Major Incident

HICG’ response was affected by a plethora of aspects, in particular expectations, previous experiences and uncertainty. Uncertainty emerged throughout the analysis, permeating all portions of HICG decision‐making and response. The following categories describe how response was perceived to be affected by previous experience from other incidents, simulation exercises and education. The categories explain how some of the factors were seen as both facilitators and barriers depending upon the circumstances.

While several factors affected response and areas in need of improvement were identified, HICGs’ overall disaster preparedness was at acceptable levels according to the majority of DPCs. The main category will be further described by the three categories and subcategories below.

### 3.2 Gaining situational awareness

This category describes the DPCs’ experience of situational awareness. To gain situational awareness, the HICG initially needed to manage the overflow of information and form the HICG. Effective formation of the HICG for a few of the hospitals was negatively affected due to a lack of trust concerning the notification system leading to time‐consuming initiatives that may have negatively impacted situational awareness and formation of the HICG.

#### 3.2.1 Deciphering the overflow of information

Disaster preparedness coordinators described barriers to gaining situational awareness due to an overflow of information and suspected disinformation. The initial challenge for HICG was that the information about the incident came from multiple sources, many of them unofficial such as social media and online news outlets. This overflow combined with a lack of information from the official source where they had expected to receive their information from, that is, the regional command caused confusion and uncertainty and made sifting through information and confirmation of details and “facts” a time‐consuming challenge.

“The amount of information caused confusion and combined with the lack of information from regional command and all this unconfirmed news, rumors started spreading, which wasn’t at all good. A lot of uncertainty…and it created stress…” (fgd)

Despite initial uncertainty, confusion and feelings of disbelief, DPCs perceived that they quickly understood the seriousness of the situation upon initial notification, and made their way to the hospital incident command centre and were able to aid in establishing the incident command.

“Initially I didn't believe it. But then things went very quickly. I quickly understood that this was serious” (fgd).

#### 3.2.2 Activating and forming the HICG

Comprehensive activation of the HICG was a priority. According to the majority of DPCs, the HICG was effectively formed, but several factors affected the activation of the HICGs. Initial insecurity was related to the lack of official information and led to full mobilization of the HICG, which in hindsight may not have been necessary. Due to regularly scheduled shift change, there was an abundance of personnel. The engagement and willingness of the personnel to aid the HICG facilitated the initial forming of the HICG.

” for our command group, some of us couldn’t get to the hospital (due to lack of transportation) so it was a good thing that there were so many people there” (fgd)

However, this was also described as a factor that amplified the confusion and stress according to the DPCs due to lack of training and education and may have led to misdirected priorities.

“A group of stand‐by physicians happened to be having a meeting in a nearby room as well as managers for logistics. These two groups see the news on social media and… take it upon themselves to go to the command center. They’re not trained in disaster preparedness and wonder ‘how do we start this? Well, we’re going to need coffee. Go get some coffee!” (fgd)

All hospitals have systems for activating and notifying members of the HICG with some utilizing an automatic notification system. However, according to a few DPCs, these notification systems were used with varying degrees of effectiveness. Activation of the HICG was impacted by insecurity about whether or not the notification systems were trustworthy with some deviating from the plan concerning the automatic system and deciding to call to all individuals as well.

“Those in charge of notifying the command group were uncertain as to how to notify us, so they decided to call each individual and confirm that they could split us up”. (fgd)

Another challenge in forming and activating the HICG was the shutdown of public transportation and a lack of available alternative forms of transportation. The majority of HICG were able to staff the various functions. However, unforeseen difficulties getting to and from hospitals for various functions forced the DPCs to find alternative solutions such as establishing other forms of contact to aid in incident management and enlisting the aid of law enforcement for security.

“People couldn’t get there. We didn’t have our incident commander, security officer, documentation unit leader”, (fgd)

### 3.3 Transitioning to management

Situational awareness is dependent upon experience, communication, and information to take appropriate and coordinated measures. DPCs described experiencing a lack of effectiveness when transitioning from situational awareness to response, citing staff overflow, and information vacuum as challenges to overcome for effective incident management. Additionally, the DPCs perceived that there was a discrepancy between expectations and unfolding events which led to uncertainty. To compensate for the information vacuum, HICGs assigned runners between the command and the emergency department (ED) and relied on information from other unofficial sources, such as ambulance personnel, police officers, and social media. Additionally, according to DPCs, effective management is affected by individual engagement as opposed to the systematic implementation of disaster training.

“The importance of this group needs to be recognized within the hospital organization so that they are allotted time for education and training”. (fi)

#### 3.3.1 Managing staff overflow

Due to the time of the incident, hospital personnel were abundant. The initial surplus of staff was in some circumstances seen as necessary and reassuring and DPCs explained that while there were nearly double the amount of staff needed, it gave a feeling of security.

“…we had double the amount of staff for quite some time, but initially I think we needed it…” (fgd)

While the surge of staff ensured that all functions of the HICG for most of the hospitals were manned despite difficulties in getting to the hospital, the surplus of staff led to crowding at the command centre and a barrier to effective management.

“When we, the HICG arrive at the command center, there’s a massive amount of people there, I’ll never forget, but what are they all doing here?? The one that had taken the role of commander, didn’t have any education whatsoever for this. He didn’t know he was supposed to remove people” (fgd)

To gain control of the overflow of staff, HICGs quickly formulated plans for staff manageability and retention by scheduling staff and providing sleeping arrangements. This allowed personnel to feel confident that they would be of use and would soon be back to aid in incident management.

” Relatively quickly we decided which ones would come back as early as 10 in the evening, personnel left more willingly when they were given an assignment and would soon be back, in some way they felt seen and needed….” (fgd)

#### 3.3.2 Struggling with Staff briefings

Even though a majority of HICGs’ staff briefings were frequent and mainly effective, several factors affecting briefings were identified, such as a lack of training of HICG members and staff overflow. The overflow of staff led to a lack of discipline resulting in inattentive staff during the staff briefings.

“there were too many of us at the first staff‐briefing, it was simply too confusing in the beginning” (fgd)

According to DPCs, effective staff briefings were facilitated when an incident commander was well trained, conversely, briefings were ineffective or absent when the incident commander lacked training or experience.

“It's easy when the incident commander is trained so they can be very constructive…”

“We had an incident commander that wasn’t trained for this, but was allowed to continue because he’s gotten the HICG up and running with assistance from all of us others, but there were no formal staff briefings” (fgd)

#### 3.3.3 Managing the information vacuum

HICGs had been trained to expect effective two‐way communication with the regional command to aid in decision‐making and response. However, the expected two‐way communication with expected sources of credible information region was initially absent. This unfulfilled expectation caused uncertainty with how to move forward, prompting HICG to rely on other sources.

“…so, we tried to contact regional command, and it was the same there, we couldn’t reach them, so our command leader just made a decision” (fgd)

“We received information from people circulating in the city and coming into the ED and the information that came to the ED was passed on to the HICGs, or from social media to the HICGs which was online (monitoring)” (fgd)

Furthermore, silence from regional command caused those in charge of communications to question whether the call‐system was functioning.

“I sat there…stressing that there was something wrong with RAKEL (emergency radio communication system in the study setting) I thought that for a long time” (fgd)

The lack of information negatively impacted the outgoing communication from the HICG with information to other vital actors at times being delayed or missing altogether.

“We had a plan for communication with the press, but we didn’t have communication between the ED and HICG…. I felt the need to go down to the ER for their sake, like a communicator…” (fgd)

### 3.4 Actions taken during uncertainty

Several factors, not thoroughly addressed in disaster plans, affected HICG performance. In this category, it is described how the experience of a real‐life MI in combination with expectations from similar training simulations led to insights and changes in disaster preparedness efforts of the HICG and the decisions and actions taken during uncertainty.

#### 3.4.1 An excessive response due to uncertainty

HICGs’ initial response to the incident was primarily described as efficient and the overall management as adequate. In hindsight, some of the DPCs expressed that hospitals’ level of response could be seen as an excessive measure. The majority of HICGs decided on the highest level of response directly after alarm. However, DPCs considered this decision to be based primarily on uncertainty as opposed to adequate situational awareness. Furthermore, DPCs expressed concerns over the ability to retain personnel as factors leading to HICGs deciding on “state of disaster” for their hospital's response.

” I’d say that we decided on “state of disaster” mainly because the other hospitals had…and because regarding staff and space. With the facts that we had; we could have stayed in partial mobilization. We would have managed, but it became full mobilization due to uncertainty”. (fgd)

DPCs expressed that disaster preparedness plans primarily are designed for “state of disaster” response. The perceived lack of protocol for the formation and staffing of the HICG for lower levels of response created uncertainty which led to declaring a “state of disaster” since this level of response activates the entire command group.

“Our plan is designed for a ‘state of disaster’ response…and what we noticed during this incident was that ‘partial mobilization’ …there was no description of who does what… so we’ve learned from this and have added a special section that has to do with “standby” and partial mobilization so there’s direction in the beginning” (fgd)

Additionally, outside pressure in the form of ED physicians’ uncertainty concerning the level of response was a stressor for the HICGs. This added to their uncertainty and factored in taking the highest level of response.

“There’s a lot of pressure from clinicians that are insecure…” (fgd)

Difficulties in gaining situational awareness were also described as a factor impacting the HICGs’ level of response.

“It has to do with situational awareness, and we are working on a way to help everyone think in the same way…to achieve situational awareness, deciding on the level of response, prioritized actions and communication and that’s something we’ve already started working on” (fi)

#### 3.4.2 Experience of balancing staff

According to DPCs, a lack of proactive decision‐making concerning the staffing of the EDs was a concern. Surgical/orthopaedic patients already at the ED before the time of the event were transferred to other sections of the ED, per disaster plans. This created an imbalance of resources for those sections according to the DPCs. As one DPC explained, those sections waited for a surge of patients who never came while other sections of the ED saw a dramatic increase of critical patients as a result of the transfer of patients.

“We had a problem there, […] those 60 extra (staff from surgical and orthopaedic sections), sat and ate pizza at the ED and then the night shift came and all the priority 2 and 3 patients were left for the night shift”. (fgd)

Most hospitals lowered the level of response in the evening, allowing the personnel from the day and evening shift to go home. According to DPCs, little consideration was given to the workload or emotional stress that night shift personnel might experience. DPCs concluded that staff should initially have been retained to aid the night shift with the extra workload and facilitating adaptation to the unusual situation.

”We could have kept at least 10 people until 24, 0100, 0200 at night, it became obvious for us, plus that they were coming in after a terror attack and were going to work night at this hospital and orientate their mindset knowing that this had just happened” (fgd)

The majority of DPCs assessed disaster preparedness as good or acceptable. However, education and training needs for the HICG were experienced as not being an established priority. This, according to DPCs makes response reliant upon the engagement of a small group of individuals, who may not be well trained.

”It’s (preparedness) going to be dependent upon individuals, unfortunately, I’d say… it’s a rather small group so that if the large portion would be activated during a Major Incident, I’d say it’s going to be dependent on the individuals and they are not particularly well trained” (fi)

## 4 DISCUSSION

### 4.1 Discussion of findings

Response and management involve strategic decision‐making, highlighting the importance of adequate decision‐making abilities (Murphy et al., 2020; Ruter et al., 2016). The results from this exploratory qualitative study identified that according to RNs in their role as DPCs, expectations, previous experiences and uncertainty affected their and HICGs’ response during an MI.

Research has demonstrated that decision‐making correlates with the overall HICG’ performance (Murphy et al., 2020). Decision‐making in health care involves some amount of uncertainty (Thompson & Dowding, 2001). Reducing uncertainty facilitates positive action and decision‐making. The DPCs experienced that when unfolding events were misaligned with expectations based on their previous training, it resulted in uncertainty. Relying on past experiences to gain situational awareness and facilitate decision‐making is a common process (Bond & Cooper, 2006). Misalignment does not inherently cause uncertainty, but uncertainty is caused either by lack of knowledge, lack of empirical knowledge, or a combination of those two (Katz, 1984). These types of uncertainty were identified during the various stages of disaster management as described by the DPCs.

Reducing uncertainty (Bond & Cooper, 2006) is vital for establishing situational awareness. The HICGs attempted to reduce their uncertainty in various ways, for example, through gathering information. A breakdown of or ineffective communication between agencies is a common challenge during MIs (Juffermans & Bierens, 2010; Turegano‐Fuentes et al., 2008). The categories “deciphering the overflow of information” and “managing the information vacuum” demonstrate factors that lead to uncertainty. Initially, HICG strived to reduce uncertainty by attempting to gather reliable information per previous training. However, the overflow of information from various unofficial sources in combination with the lack of communication from official sources, made it difficult to achieve situational awareness and reduce the uncertainty. To compensate, other known strategies for reducing uncertainty, for example, rational reasoning and intuition‐based decision‐making (Shortland et al., 2018) were used. One of the DPCs intuitively took action by implementing a runner between the ED and incident command. Another DPC combined information from other sources, such as online media, news outlets and people coming into the ED with rational reasoning, to reduce uncertainty and facilitate positive action.

Staff overflow was another challenge for HICGs. This challenge mirrors previous studies that concludes that health care personnel’ willingness to aid in disaster response is high (Baack & Alfred, 2013; Fung & Loke, 2013; Nasiri et al., 2019.

The willingness by health care personnel to aid in disaster response is also associated with many barriers. In this study, DPCs experienced staff overflow as both an obstacle and a success for effective response. Staff overflow was primarily a hinder when attempting to gain an overall control of the situation including dissemination of information. However, excess staff also facilitated the reduction of uncertainty once the action was taken to utilize excess staff to fill vacancies or to proactively plan for the relief of staff and replacement.

Experience and expectations were recurring factors affecting uncertainty and response. To train and evaluate HICG disaster preparedness, simulation exercises are often used. The hospitals in the study had recently participated in terror‐related simulations. According to the DPCs, HICGs responded per experiences from past training and the expectations created uncertainty when unfulfilled. However, while reducing uncertainty can facilitate decision‐making and response, in light of these results, the opposite can also be true, that is, decision‐making can reduce uncertainty (Katz, 1984). HICGs decision to scale up hospital response to the highest level of response was to reduce uncertainty by increasing competence. ”State of disaster” primarily ensures that all sections of the HICG are activated. This in turn may increase group competency, knowledge and experience, facilitating positive action.

DPCs expressed that disaster preparedness was adequate but that response for future MI is dependent upon individual engagement as opposed to systematic implementation of training and education. This study identified that in addition to this, uncertainty permeates HICG’ response. Suboptimally trained management could have negative implications for patient outcome. Proficient decision‐makers often have experience within their area of expertise from which they can draw from previous situations to aid in response (Bond & Cooper, 2006). The low number of MIs in the study setting limits the amount of real‐life experience, underscoring the importance of training. While simulations and training positively impacted structural and process knowledge, the ability to reduce uncertainty appears to be an area that should be targeted in future training programs.

### 4.2 Limitations/Methodological considerations

The small number of participants (*n* = 6), and the total amount of time in this study can be seen as a limitation, something that was considered and discussed in the research team when designing the study. However, Malterud et al. and Vasileioue et al. suggest that information power as opposed to the number of interviews or reaching saturation is more relevant (Malterud et al., 2016; Vasileiou et al., 2018). Information power is reliant on aim, sample specificity, quality of dialog and analysis method (Malterud et al., 2016). Even though the sample is small, these participants represent 100% of those total population (one DPC from each of the six hospitals in the area) and therefore they are the only ones who can answer the specific aim of this study. The follow‐up interviews were allotted 60 min. However, the participants did not have more to elaborate on from what they had already discussed in the FGI, which made the follow‐up interviews short. This suggests that the participants had expressed everything they could share on the subject, which is another criteria for information power and a strength of the study (Malterud et al., 2016).

While the empirical support as assessed by number of participants, and interview minutes could be seen as a limitation, the specific aim, specificity of participants, richness of dialog and analysis strategy indicate that information power was sufficient for this particular study.

One of the DPCs did not partake in the interviews due to their being a part of the research team. To avoid data collection bias (Gerrish & Lacey, 2010), this DPC was replaced by an assistant DPC for all of the interviews. While this could be seen as a limitation, this assistant DPC fulfilled the inclusion criteria and was an adequate replacement for the DPC, which ensured that all six hospitals were adequately represented.

Preunderstanding in combination with open‐ended questions can help extract data (Marshall & Rossman, 2016) but can also lead to biased interpretation (Polit & Beck, 2021). JM and MR conduct research on the subject matter and have a prior understanding of the participants. To minimize bias, a facilitator without preunderstanding of the subject matter or the participants but with extensive knowledge in qualitative research (MJ) was included in all phases of the study. In addition, to further ensure trustworthiness, investigator triangulation (Denzin, 1978) was used with a total of four researchers for the analysis; In addition to JM, MJ, MR, a researcher in emergency nursing with experience in content analysis, AH was included.

This study contributes with knowledge about the challenges DPCs and HICGs face during an MI. The steps taken to increase trustworthiness and information power provide rich and detailed insights, presenting factors affecting HICG’ response, and may therefore facilitate transferability of the results to similar contexts.

## 5 CONCLUSION

According to DPCs, experience, expectations and uncertainty are factors that are crucial for effective disaster management and to improve patient outcome. For positive action, it is necessary to reduce uncertainty and therefore, reducing uncertainty is essential for effective disaster response since it is future‐oriented. Education and training based on realistic scenarios which focus on the command's ability to reduce uncertainty could improve management skills and positively affect patient outcome.

## CONFLICT OF INTEREST

The authors declare that they have no competing interests.

## AUTHOR CONTRIBUTIONS

Conceptualization: JM, AR, MR, LK. Methodology, reviewing, interpretation and validation: JM, MJ, AH, MR. Data collection: JM, MJ. Initial data analysis, interpretation, conclusions, and original draft preparation: JM. Writing, reviewing and final draft: All authors. All authors approved the final manuscript.

## Data Availability

Due to the possibility of participants’ privacy being compromised, the data are not publicly available. However, the data that support the findings of this study are available on request from the corresponding author [JM].
